# Dental Education: A Retrospective and Prospective View

**Published:** 1904-08

**Authors:** John S. Marshall

**Affiliations:** San Francisco, Cal.


### DENTAL EDUCATION: A RETROSPECTIVE AND PROSPECTIVE VIEW.[1]

BY JOHN S. MARSHALL, M.S., M.D., SAN FRANCISCO, CAL.

"We will not anticipate the past, our retrospection will be all of the future."

It is my purpose in this short paper to take a hasty retrospective glance at the history of dental education in the United States, that I may hold before you for a moment a picture of progress that has seldom been equalled in the history of educational advancement, and then to change the view and hold before you another picture, a prospective view, the realization of which will be no more difficult to us and our children than was that of the first picture to our fathers and ourselves.

"Past and to come seems best; things present worse."

Shakespeare never wrote a truer line than this, for, if we were satisfied with the present, there would be no progress in the future. Satisfaction with the present brought decay and ruin on the ancient Egyptians and on the Roman Empire.

### A RETROSPECTIVE VIEW.

We learn from history that the art and science of surgery is an outgrowth or evolution of the tonsorial art. Two hundred years ago the barber was also the surgeon. His functions were to shave and cut hair; to bleed the prince or the pauper; to bind up wounds and set broken bones. The striped red, white, and blue pole which stood before his shop indicated that he was ready on occasion to bleed his patron, bind up his wounds, or set his broken bones. The term applied to him was barber-surgeon. His social position was that of a barber, a person to be petted on occasion, or insulted or kicked at the caprice of those of higher social position. To-day his learning and skill have placed him on the highest pinnacle of fame by reason of which kings and emperors

[1] Read at the fifty-fifth annual session of the American Medical Association, in the Section on Stomatology, Atlantic City, June 7 to 10, 1904.

have made him their confidant, and freely and without fear have placed their lives in his keeping.

Dental surgery was likewise evolved from the art of the barber, the tinker, and the village blacksmith. A little over sixty years ago there was no such institution as a dental college, no such organization as a dental society or even a journal devoted to the interests of dental surgery. At that time the only means of education in dentistry was by the old system of apprenticeship. The dentist in those days had no social or professional standing; he was envied by the barber and looked down on by the doctor of medicine. To-day he is regarded as a member of a noble profession, standing shoulder to shoulder with the physicians and surgeons and dividing with them the honor of adding to the comfort and longevity of the human race by reason of their learning and technical skill.

The evolution of the surgeon, from the barber to the scientist, required several centuries, while the evolution of the dentist from his low degree to his present honorable position has been accomplished in as many decades. This has been wrought partly by the energy and forethought of the pioneers in the profession, but principally by the more favorable environment which surrounded the infant profession.

In these days of the rapid spread of knowledge and learning, the development of the arts and sciences and of the inventive faculties, the ignorance, prejudice, and bigotry of the race have been swept away and all men and all movements, either social, political, or scientific, are given an opportunity to develop, each standing or falling according to its merit. Surgery in its early struggles had to fight against the ignorance of the people, the prejudice of the scholars, and the bigotry of the church, and little progress was made until the beginning of the last century. The discovery of the microscope, of vaccination, anæsthesia, and antiseptics made it possible for medicine, surgery, and dentistry to reach their present high development, which is still progressing and must continue to advance in the future.

Dental education may be said to have had its beginning in the organization of the first dental college. This institution, as we all know, was incorporated and established in the city of Baltimore, Md., in the year 1840. Its entrance requirements were simply a desire to learn. Its curriculum was anatomy, physiology, chemistry, development of the teeth, principles of operative and mechanical dentistry, and laboratory instruction; yet with this meagre course of instruction, it developed some of the brightest minds that have been found in the ranks of the profession. Its requirements for graduation were for practitioners of five years' standing, one course of lectures of about four months; for students, two courses.

A reference to the text-books of this period will show how circumscribed and narrow was the field of study, when compared with the text-books of to-day and the requirements for entrance and graduation from our best dental colleges.

With the organization of other dental colleges in various parts of the country, a rivalry sprang up between them, and as the profession advanced in scientific knowledge the curriculum was broadened and the courses extended to five months in each year.

For many years thereafter little or no advancement was made either in the course of instruction or the length of the college year. Hydraheaded commercialism had entered into the schools, taken them by the throat, and well-nigh strangled them, for the great aim in most of them had become large classes and a substantial dividend at the end of the year to be divided among the incorporators.

The organization of dental societies, local, State, and national, and the establishment of dental journals, which followed very soon after the incorporation of the Baltimore Dental College, and through the succeeding twenty years, were the most potent elements in arousing the colleges from the stagnation into which they had fallen through the commercial spirit. The might and power of these influences are still exerted for the uplifting and stimulating of the best impulses in education, professionalism, and ethics. To the dental societies and the dental journals the colleges and the profession at large owe much of their success and scientific advancement.

The regulation of the practice of dentistry by the passage of laws for this purpose, by several of the older States, was an advance step of great moment to the interests of higher dental education. These laws recognized dental surgery as a department of medicine, and for the first time in history it was raised to the dignity of a professional calling. The public had heretofore looked on the dentist as a mechanic, spoke of the trade of dentistry, referred to his office as his shop and his operations as jobs. And, although the dental surgeon was the possessor of the honorable degree of D.D.S., conferred *en course,* many persons addressed him by his title in the same spirit that they spoke of the barber as "professor," or the chiropodist as "doctor."

Time, however, changed all this, and the public, by degrees, came to look on dentistry as a professional calling worthy of the best minds and greatest talents. As a result of this change of status in the minds of the public, the dental colleges of this period were overcrowded with students. The demand for more schools seemed imperative, and, as a natural result, many were organized, too many for the best interests of education. Competition of a commercial character, even more strenuous and active than before, took possession of many of the colleges, and some of the weaker schools, feeling that they were being crowded to the wall, opened their doors to any who could pay the fees, promising to graduate them at the end of two courses of lectures. In many instances students were graduated after one course of lectures, and, to our shame be it said, diplomas were issued in some instances to men who had only attended a sufficient number of lectures to identify themselves with the graduating class.

The courses of instruction in the dental colleges of this period were so diverse in subjects and study requirements that students had great difficulty in passing from one college to another, as the faculties of the different colleges often refused to give credit for work done in other institutions. This state of affairs became so unsatisfactory to the colleges and students that several of the leading schools issued a call for a conference of all the dental colleges, for the purposes of harmonizing the courses of study and arranging for comity between the schools and such other matters as should be of mutual benefit to all concerned. This conference resulted in the organization of the National Association of Dental Faculties.

The organization of this Association marked a great epoch in the history of dental education, for through its deliberations and established rules it has exerted a tremendous moral influence over the colleges, both within and without the Association. It has kept the colleges within the Association up to its prescribed standards for the admission and graduation of students, while those on the outside have been compelled by the State examining boards to seek admission to the Association and place themselves in harmony with the requirements of its educational standards. It has brought order out of chaos, in dental teaching; it has harmonized the courses of instruction and has stopped the practice of granting degrees *in absentia.* It has made it possible for students with proper credentials to finish their course of instruction in any college that they may elect. It advanced the requirements for graduation from two to three years. It established an educational entrance requirement and gradually raised this requirement from nothing to a certificate of having passed the examination of the second year in high school. It has introduced technic teaching in operative and prosthetic dentistry. It has improved and extended the curriculum from time to time by adding such important subjects as physics, embryology, general materia medica and therapeutics, general pathology, bacteriology, comparative dental anatomy, orthodontia, general and oral surgery.

Up to one year ago the minimum preliminary requirements for entrance to our dental colleges was a certificate of having passed the studies of the second year in high school, and for graduation, three full courses of instruction of from seven to nine months in each year.

Until three years ago this was the same standard as that required by the medical colleges. At this time the medical courses were extended to four years and the minimum entrance requirement was advanced to a diploma from a four-year high school. A few of the better class of medical schools now require that the student shall have passed the studies of the sophomore year in liberal arts or natural science, while one insists on the bachelor's degree.

At the present time the minimum preliminary requirements for entrance to the dental colleges having membership in the National Association of Dental College Faculties is a certificate admitting the student to the third year of a high school or its equivalent, while that for graduation is four annual courses of instruction of not less than seven months in each year. This, it seems to me, is a record of which the dental profession need not feel ashamed, and yet it should not felicitate itself too much over its progress, for much greater advancement might have been made in educational matters if the colleges could have been united in their work from the beginning.

A PROSPECTIVE VIEW.

The second picture, or the prospective view, must necessarily be one made up largely of personal opinions and impressions, but nevertheless opinions and impressions bred of many years of experience and general observation.

The great need of the profession to-day and for the future is, in my opinion, higher preliminary education and broader and fuller instruction in those fundamental sciences which make up the sum total of a dental education. The profession has done well in the past, but it cannot afford to relax its efforts or to take a backward step, now or ever.

For many years the minimum preliminary educational requirements for entrance to the dental colleges were the same as for matriculation in the medical schools, but at the present time they have fallen somewhat behind. This, in my judgment, is a mistake, and it should be rectified at the earliest opportunity.

The requirements for matriculation and graduation in dental surgery should be in no way inferior to the requirements of the medical schools. The courses of instruction in the fundamental sciences should be the same, and during this period the medical and dental students in the great universities should be taught together, and no distinction made between them. By this plan better and more thorough work can be done and at much less expense. This saving of expense would make it possible to employ teachers, at an adequate salary, who would give their whole time and attention to teaching and original investigation, and as a consequence the teaching would be of a higher grade and the work of the student would be more enthusiastically performed.

What we need to-day and in the future is wide-awake teachers for the fundamental sciences, teachers who are full of their subject and who can impart knowledge in such a fashion that the student cannot help being interested in these, to him, otherwise dry subjects. If the teachers of anatomy, physiology, chemistry, etc., are not enthusiastic in their work, the student soon loses interest in them, and turns to the more practical subjects to which he often gives time that could be spent to better advantage on the fundamentals, as he finds, to his chagrin, in after years.

In my work as an examiner for the Dental Corps of the United States army, I have found that the majority of the candidates who have presented themselves for these examinations were deficient in preliminary education and in those subjects usually denominated as the fundamental sciences. In the practical departments they were, as a rule, quite well prepared. The best marks were usually obtained in operative and prosthetic dentistry, crown- and bridge-work, and orthodontia. In fact, many of them were good jewellers, but lacking in that knowledge which marks the truly professional dentist. This is not the fault of the curriculum, for with the subjects that now comprise the courses of study in our better dental colleges the conscientious, hard-working, well-prepared student will receive an excellent professional education.

The most serious difficulties which the teachers of the fundamental sciences experience in their work is the lack of time properly to cover the subjects and mental deficiency on the part of a large number of students in every class, whose minds have not been sufficiently trained and disciplined by previous study readily to grasp or comprehend the sciences which they are endeavoring to teach. As a consequence, the instructor is obliged to suit his teaching to the comprehension and ability of these students, while those of higher educational requirements, who could progress much more rapidly had they the opportunity, lose interest in the subject for want of a stimulus to study. This fault can only be corrected by insisting on a higher grade of preliminary educational requirements, and I most sincerely hope that the Faculties Association will not only raise these minimum requirements to graduation from a four years' high school or its equivalent, at its next session, but will recommend the completion of the sophomore year or a full academic training in the liberal arts or natural sciences as the proper preparation for the study of dental surgery. It is possible at the present time for students of medicine to procure the bachelor's degree in natural science and the degree in medicine by combining the two years' work of the junior college with the four years' work of the medical school. The University of Chicago, the University of Michigan, the University of California, and Cornell University now offer such courses, both degrees being conferred on the candidate after the completion of his full medical course.

This is a movement in the right direction, and I can see no reason why the universities having dental colleges should not offer the same inducements to their students in dental surgery. With such a class of students the work of the instructor would be a joy, while the profession would gain very greatly by the addition to its ranks of a class of more highly educated practitioners.

A young man who has received a college training has usually been taught the principles of social and political ethics, and is therefore better prepared to understand and appreciate professional ethics. Such a young man is generally imbued with higher standards of conduct and of living. He recognizes the ethical rights of others and demands that he be accorded the same rights. Such men are seldom guilty of conduct that brings reproach on their profession. They are seldom found in the ranks of the advertising quacks or those who selfishly patent a remedy, an instrument, or an improvement in methods, which would be of benefit to suffering humanity, forgetting, or not caring, that nearly every remedy they employ for the cure of disease, every instrument they use, or operation they have been taught to perform was thought out and perfected by some member of the profession who preceded them, and who freely and unselfishly gave these to their brethren for the benefit of suffering humanity. The profession needs more of this unselfish, better-educated, truly professional class of men. It needs to stamp out the commercial idea and install in its place the true professional spirit which places our duty to our patients and the State first, leaving the question of the fee for future consideration.

The lengthening of the college course from three to four years will call out many differences of opinion as to how the extra year shall be employed. Those who teach the practical branches will want to extend instruction in operative and prosthetic technics, while those who teach the fundamental sciences will ask for more time and more thorough courses. The latter, to my mind, is at present the most important side of the question, and it should be given careful and conscientious consideration, for the future of the profession depends on how it is decided.

To obtain the highest degree of instruction the teacher must be a specialist in his department, and with the financial inducements that can be offered by the average dental college such teachers for the fundamental sciences are difficult to obtain.

Specialists in the practical subjects of dental surgery are plentiful and, as a consequence, this part of the training of students leads the theoretical and the scientific.

The student is naturally attracted to those subjects on which he thinks he can see that his future financial success as a practitioner depends. He is, therefore, inclined, through mental shortsightedness and inexperience in the profession, and from his inability to comprehend its responsibilities and his duties to his patients and the State, to neglect those subjects which are of the greatest importance in the development of scientific dentistry, and hence to the welfare of his patients and the best interests of the State. He therefore devotes his energies to those departments which appear to him to promise the largest and most speedy returns in money for the time and study spent on them.

We are a nation of buyers and sellers. Commercialism dominates our people. Trade and trade interests rule the nation. How, then, can we find fault with the student if he partakes of this spirit of commercialism that he can see everywhere around him, that he reads in the newspapers, hears talked on the streets and in the parlor, sees in the bitter competition of some professional men, and cannot help but know exists in the management of many institutions of professional learning?

The best interests of higher dental education demands that the management of these schools should be taken out of the hands of the individual or the teaching faculties and placed under the control of the State, or of the trustees of our great universities, for the reason that the majority of the dental schools to-day are still per force commercial institutions, dependent on the fees of students and the profits of the infirmaries to maintain them and pay the teachers and demonstrators. Just as long as commercialism exists in our system of dental education the best results can not be obtained. The interests of the students should be paramount, but this interest is often obscured by holding the dollar too close to the eye. It is marvellous how small an object will obscure our vision if we but hold it close enough.

The first dental college was organized on the commercial basis, and I do not know of a single dental school, even those under State control, that are entirely free from some form of commercialism, for in all of them the fees of the students, or the receipts of the clinics, bear a more or less important relation, either to the salaries of some of the professors or demonstrators, to the buildings they occupy, or to the apparatus or furnishings.

In most of the State institutions the appropriations are too small properly to conduct the schools, while in the colleges connected with the large endowed universities no endowments have been made to the dental schools. They are thus left in a measure to "work out their own salvation," in many cases "with fear and trembling," as I know from personal experience.

We could wish that the legislators of the States having professional schools in their universities would appropriate for their support with a more liberal hand; and that good people with fortunes they cannot spend and do not know where to bequeath, would endow the dental colleges and place them above the degrading necessity of catering to the commercial spirit, and entering students who have not a proper educational qualification to begin the study of dental surgery, or who are deficient in that peculiar aptitude for a professional life, through which alone they can hope to develop practitioners who will be an honor to the institutions that are responsible for their education.

I have confidence to believe the day is not far distant when all of the dental schools will either be liberally provided for by the State or abundantly endowed through the munificence of a Rockefeller or a Carnegie. The American people are liberal and just towards all educational efforts, and whenever they are convinced that the best interests of the citizen and the State demand that they render financial assistance to any individual enterprise, it is usually forthcoming.

Another serious drawback in our system of education is the failure to provide advanced standing in the dental course for students who have taken the bachelor's degree in liberal arts and sciences.

These students have had a sufficient amount of didactic teaching and practical laboratory instruction in chemistry, botany, morphology, physiology, zoology, comparative anatomy, etc., to more than equal the work of the freshman class in the dental schools, and under these circumstances their higher preliminary qualifications should be recognized by advancing them to the sophomore class of the dental course. By adopting a policy of this character ambitious students would be encouraged to complete their academic training before entering on the study of dental surgery.

As the matter now stands, the college graduate is placed on the same footing as the student whose qualifications are only those of a second year course of a high school, and the extra six years of study pursued by him in the languages, mathematics, literature, history, and the sciences count for nothing. This is placing a premium, I was about to say, on ignorance, but I will modify the statement and say, on the minimum requirements for matriculation, and thereby turns away many young men from the doors of the dental schools who would have been an honor to the profession.

I am constrained, also, from my sense of justice, to make another suggestion,—namely, on the matter of advanced standing for medical graduates. Since the adoption of the four-year course for medical and dental colleges there would seem to be no good reason why medical graduates who have taken a full four years' course should not be advanced to the junior grade of the dental colleges.

The four years of training of the medical graduate, preceded by a full four years' high school course as a minimum matriculation requirement, is certainly equal to the freshman and sophomore courses of study in the fundamental sciences as taught in the dental schools, preceded by only two years of the high school course as a minimum entrance qualification. It would therefore seem no more than a just recognition of the scientific work performed that he should receive such advanced standing in a dental course. If such students, devoting their full time and energy to the study of the purely dental subjects for two years, cannot make as good operators as their fellows who entered with the minimum qualifications and have spent four years upon the dental course, then the value of higher education as a prerequisite for professional study is a myth and a snare.

Let us look at this matter in a reasonable light and place ourselves on record as favoring the proposition of giving due credit for scientific work performed by candidates for matriculation who hold the B.A., B.S., or M.D. degree from colleges and universities of recognized repute. Scientific knowledge is of as much value to the dental surgeon as it is to the physician and the surgeon, for without the employment of this knowledge in his profession, the dentist is nothing more than a handcraftsman.

